# Supplementary material for: Optically-generated focused ultrasound for noninvasive brain stimulation with ultrahigh precision
Source: Light Sci Appl. 2022 Nov 3;11:321. doi: 10.1038/s41377-022-01004-2 (PMC9630534; doi:10.1038/s41377-022-01004-2)
Supplement: Supplementary file 1 — Supplemental Material [file 41377_2022_1004_MOESM1_ESM.docx]

Supplementary Information for

**Optically-generated Focused Ultrasound for Noninvasive Brain Stimulation with Ultrahigh Precision**

Yueming Li, Ying Jiang, Lu Lan, Xiaowei Ge, Ran Cheng, Yuewei Zhan, Guo Chen, Linli Shi, Runyu Wang, Nan Zheng, Chen Yang*, Ji-Xin Cheng*

*Corresponding author. Email: jxcheng@bu.edu (J.C.); cheyang@bu.edu (C.Y.)

**This PDF file includes:**

Fig. S1 to S13

Legends for movie S1 to S4

**Other Supplementary Materials for this manuscript include the following:**

Movie S1 to S4


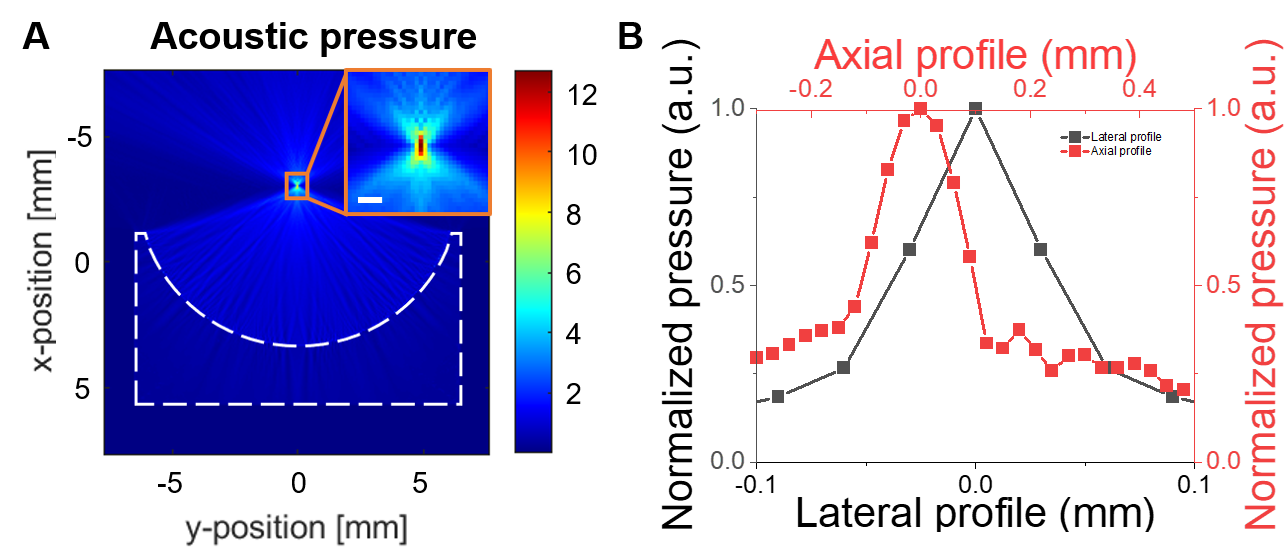


Fig. S1. Results of a 2D simulation of the acoustic field generated by SOAP with a NA of 0.95. (A) The acoustic field generated by SOAP in k-wave simulation with a NA of 0.95. Dashed line: the position of SOAP. Inset: zoom in at the acoustic focus. Scale bar: 200 µm. (B) The lateral (black) and axial (red) profiles of the generated acoustic field was plotted.


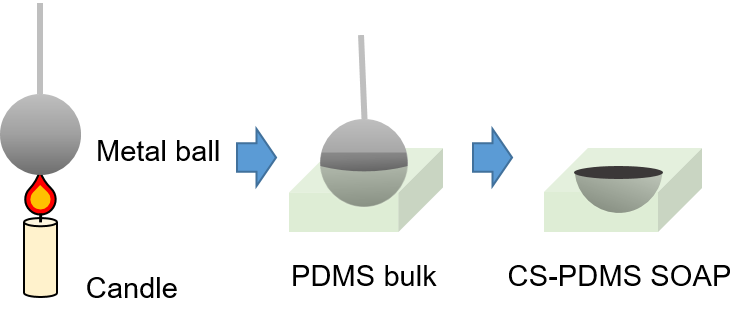


Fig. S2. The fabrication process of CS-PDMS SOAP.


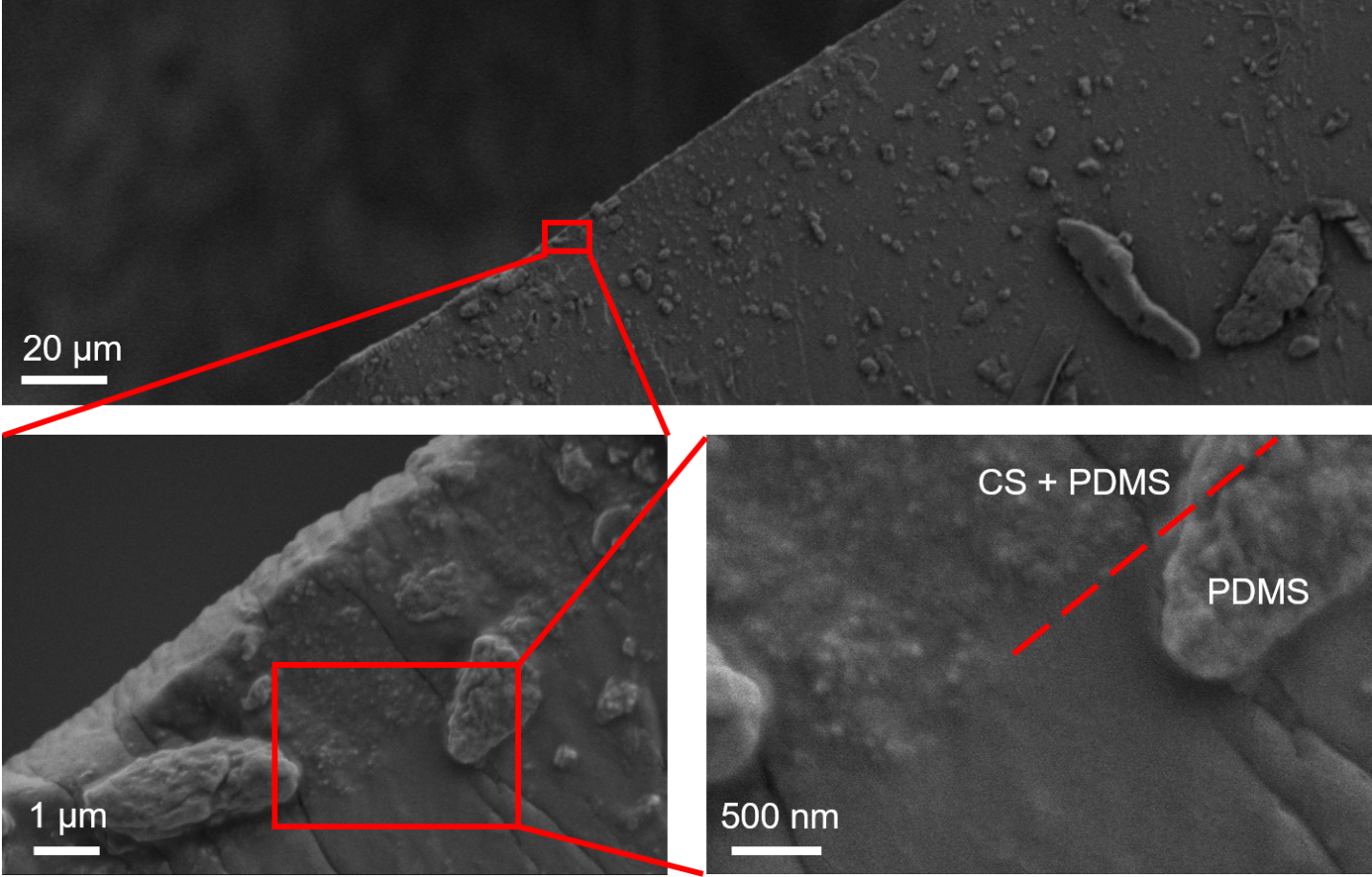


Fig. S3. SEM images of candle soot and PDMS mixture. The dashed red line separated candle soot and PDMS mixture region and pure PDMS region. The mixture region has a rough surface with embedded CS particles. The pure PDMS region has a smooth surface.


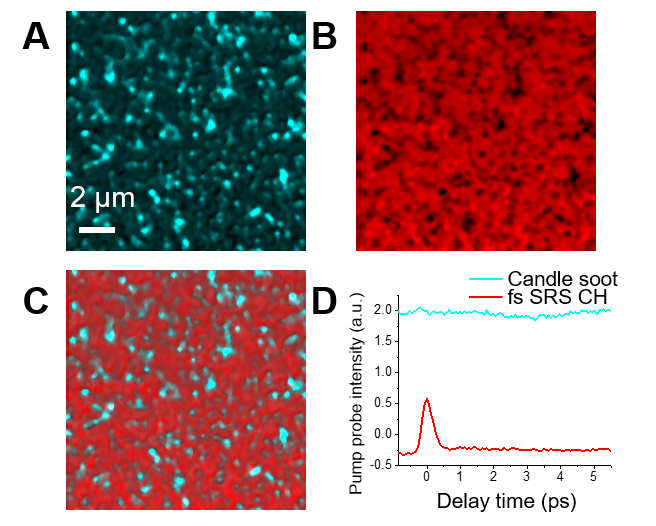


Fig. S4. SRS and photothermal images of PDMS and CS in a matrix. (A) The photothermal signal from CS reveals its distribution in the CS-PDMS mixture. (B) The SRS signal from the C-H bond in PDMS reveals its distribution in the CS-PDMS mixture. (C) The merged composition map of the CS-PDMS. (D) The pump probe intensity over tuned delay in the CS-PDMS mixture. Cyan: the photothermal signal from CS. Red: the femtosecond SRS channel for the C-H bond signal from PDMS.


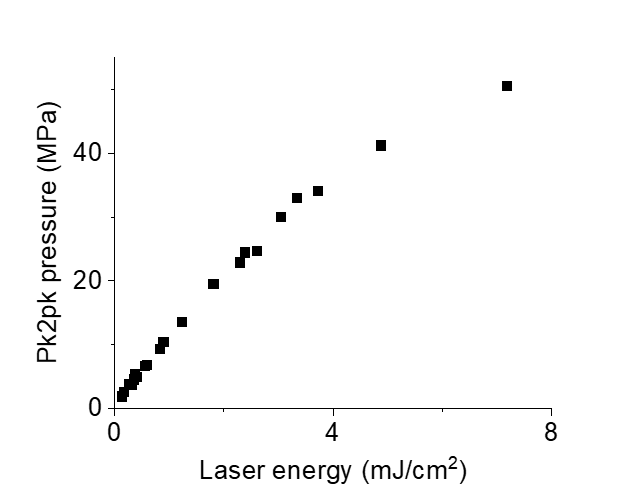


Fig. S5. The peak to peak ultrasound pressure generated by varying the input laser pulse energy. The signal was directly measured with a 75 µm hydrophone. The fitted line is *y* = 8.51**x* (*R*^2^ = 0.9754).


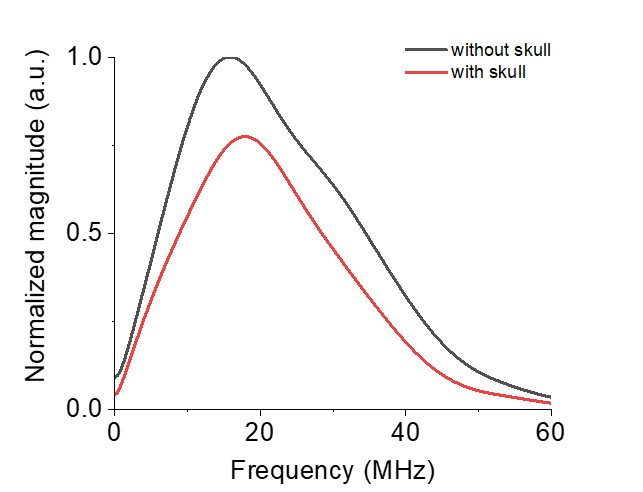


**Fig. S6.** **Frequency spectrum of OFUS without and with a piece of mouse skull.** The data were normalized to the peak amplitude of the signal without the presence of the skull at the focus.
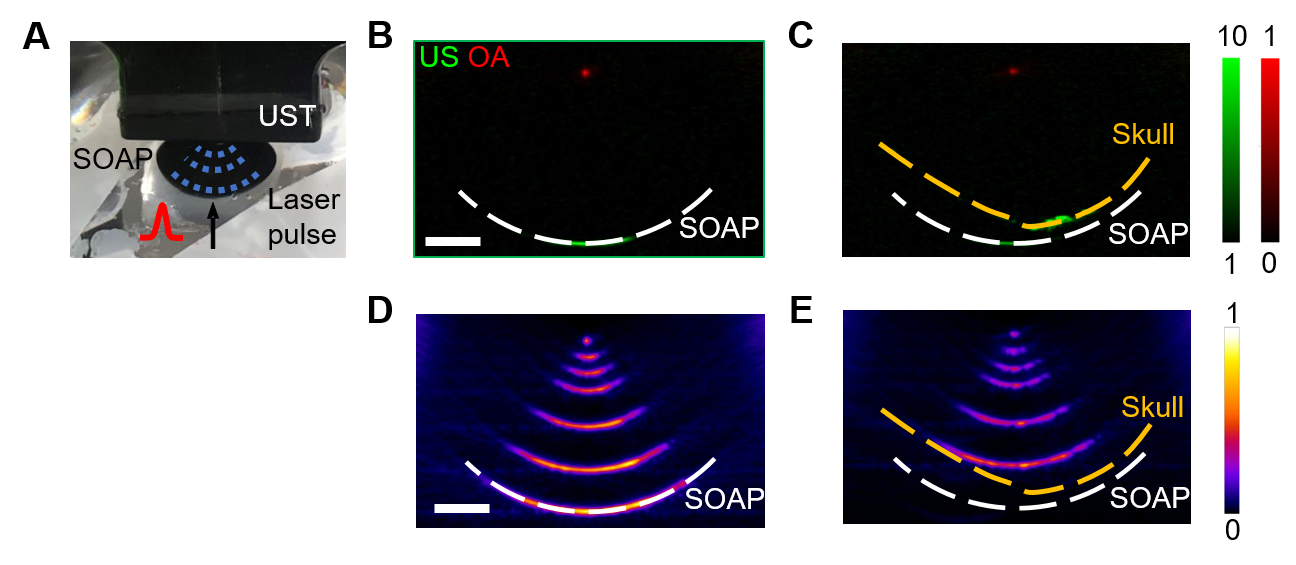


**Fig. S7.** **The visualization of the ultrasound propagation with an OAT system.** (**A**) The photo of the experimental setup of OA imaging. A pulsed laser (red) illuminated OFUS from bottom and the OA signal (blue) was collected by an ultrasound transducer array (UST) from above. (**B, C**) The merged image of ultrasound signal (green) and optoacoustic signal (red) without and with a piece of mouse skull. The optoacoustic signal is the ultrasound focus that generated by OFUS. White dashed line: OFUS. Yellow dashed line: a piece of mouse skull. Scale bar: 2 mm. (**D, E**) OFUS-generated ultrasound propagation without (**D**) and with the skull (**E**). Image scaled to propagation without skull. White dashed line: SOAP. Yellow dashed line: a piece of mouse skull. Scale bar: 2 mm.


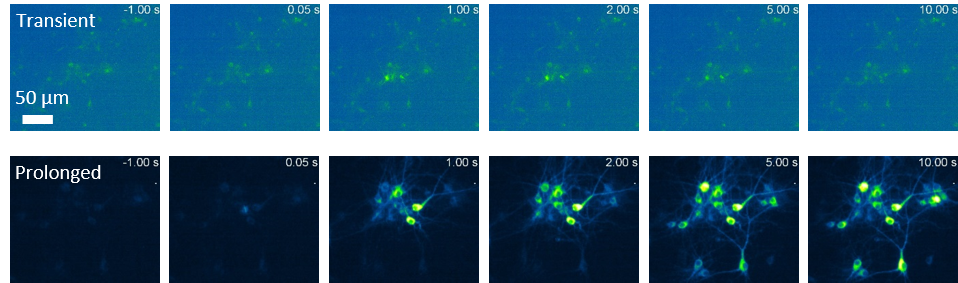


**Fig. S8.** **Representative time lapse images of the transient and prolonged cell response.**
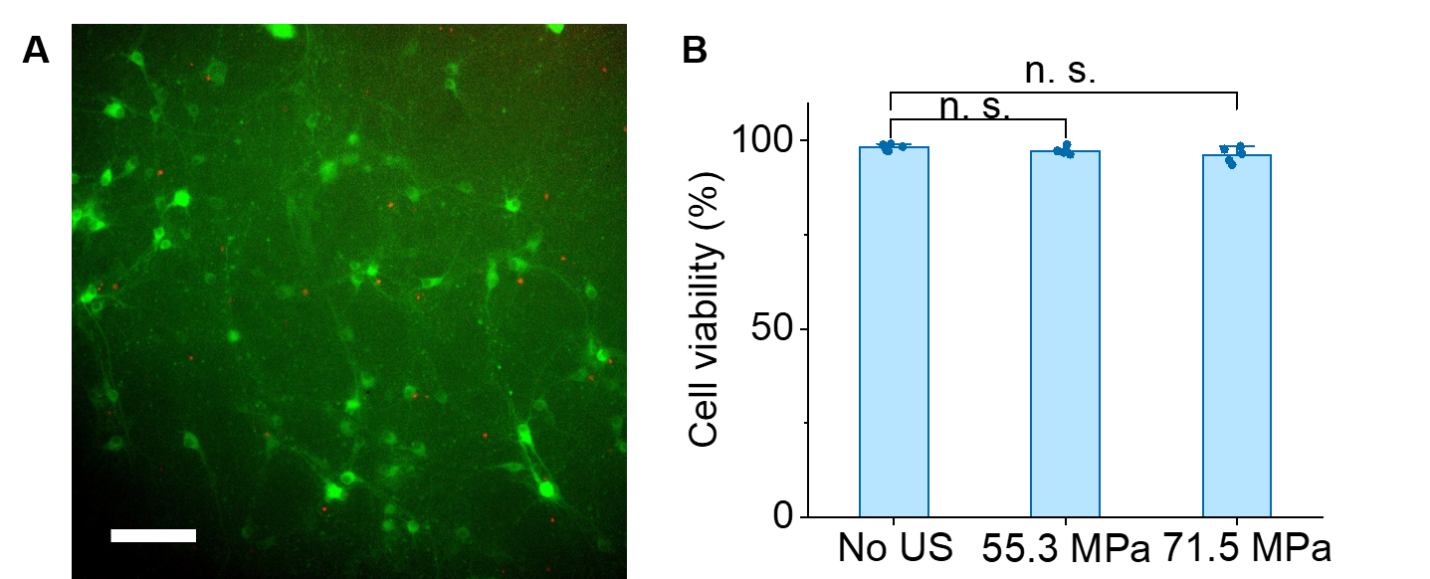


**Fig. S9. The viability study of neurons after the repeated stimulation.** (A) A representative image of the live and dead cell staining after the repeated stimulation with a peak to peak pressure of 55.3 MPa. Scale bar: 100 µm. (**B**) The statistic of the cell viability. n.s., not significant (*p* > 0.5).
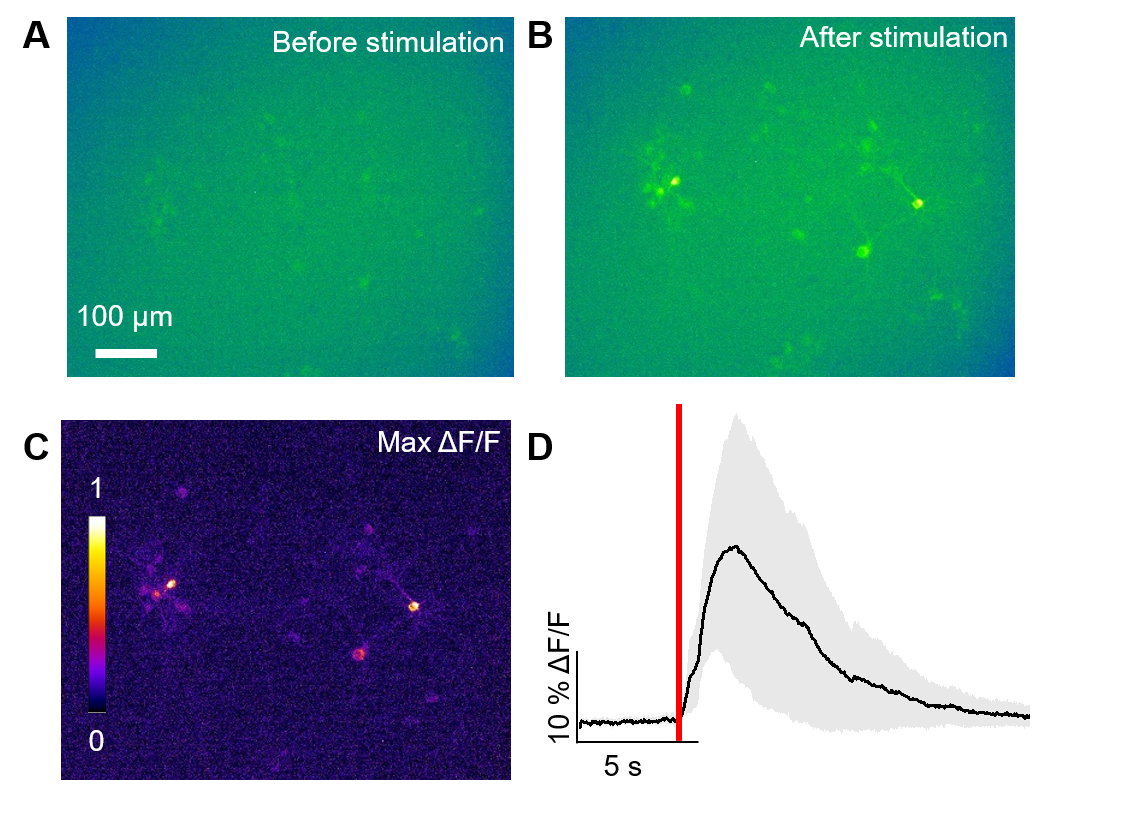


**Fig. S10.** **Neurostimulation with 20 MHz focused ultrasound transducer.** (**A**) Fluorescence images of before and (**B**) after 20 MHz focused ultrasound stimulation. (**C**) Max *ΔF/F*. (**D**) The averaged calcium signal of stimulated neurons.
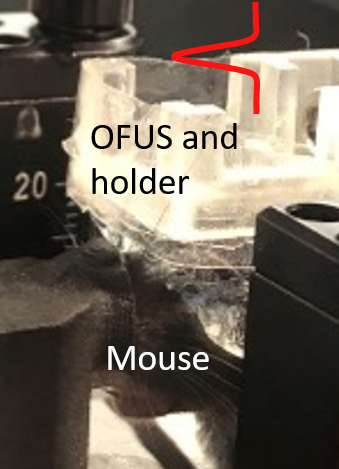


**Fig. S11.** **A photo of the experimental setup of OFUS stimulation *in vivo.***

**
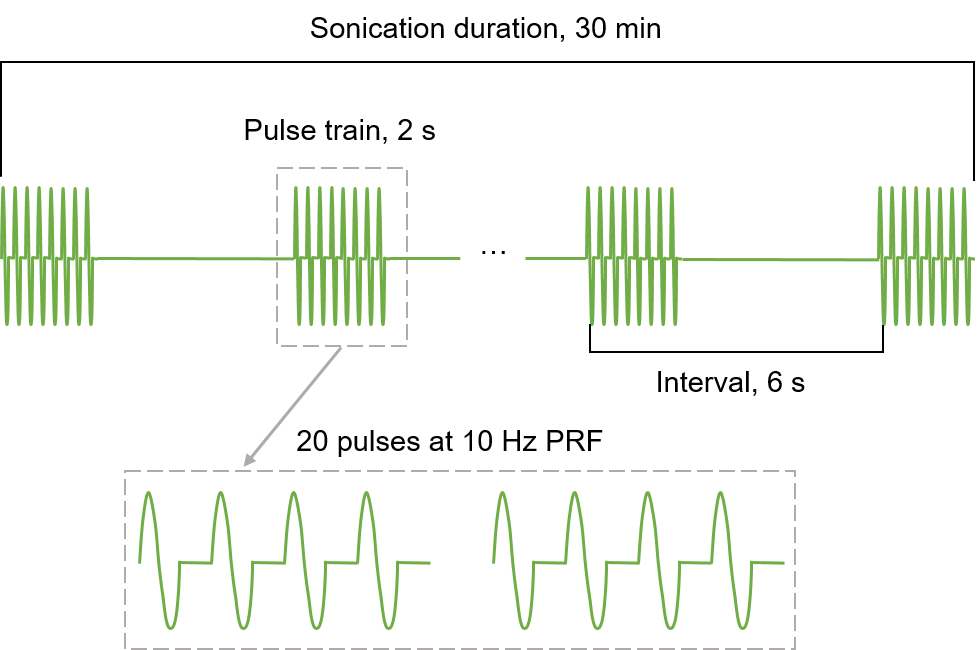
**

**Fig. S12. The ultrasound pulse train diagram for OFUS stimulation to express c-Fos.**


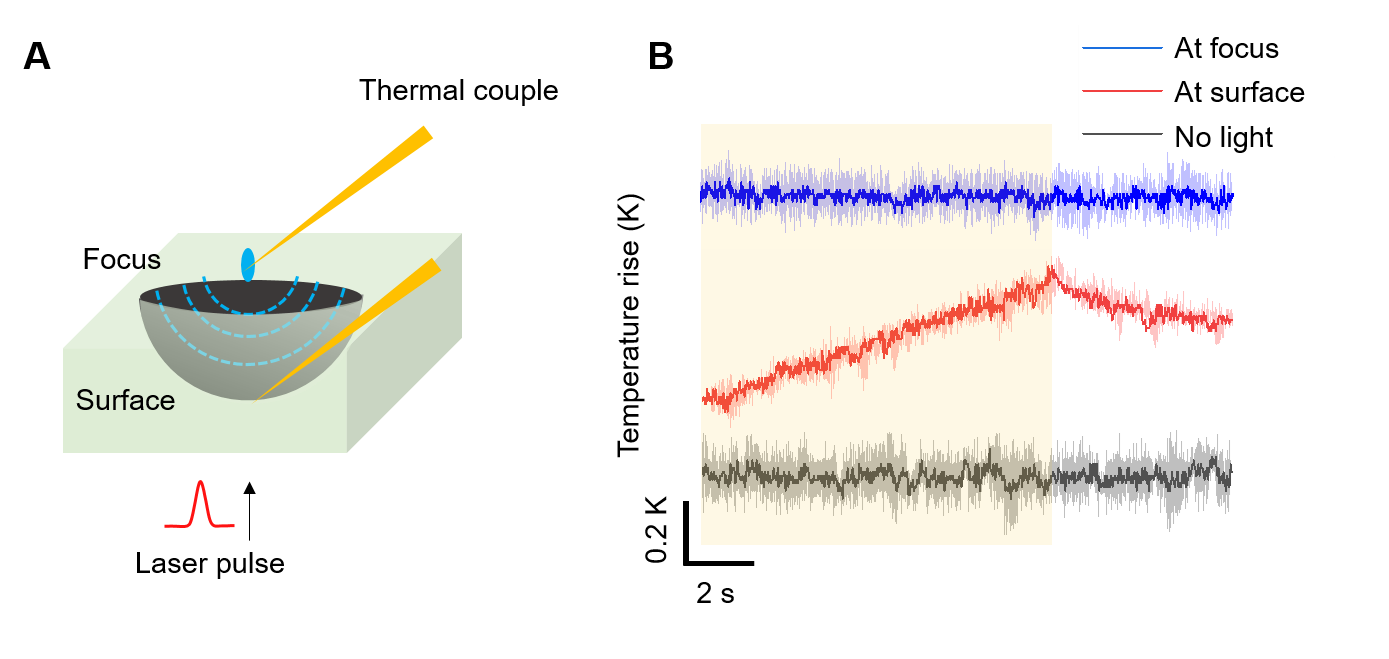


**Fig. S13.** **The temperature profile of OFUS.** (**A**) The experimental setup of the temperature measurement. (**B**) The temperature profile recorded for 15 s. Yellow box: laser on.

Movie S1. The transcranial ultrasound propagation. The propagation process of ultrasound from SOAP surface to focus and forward. Scale bar: 2 mm.

Movie S2. The visualization of OFUS focus with florescent beads. The motion of fluorescent beads indicates the location of OFUS focus. White dashed circle: OFUS focus. This movie is accelerated by a factor of 5.

Movie S3. Direct OFUS stimulation of neurons with a single cycle at a pressure of 29.8 MPa. This movie is accelerated by a factor of 5.

Movie S4. Transcranial OFUS stimulation of neurons with a single cycle at 57.0 MPa. This movie is accelerated by a factor of 5.
